# Validation of Exposure Visualization and Audible Distance Emission for Navigated Temporal Bone Drilling in Phantoms

**DOI:** 10.1371/journal.pone.0041262

**Published:** 2012-07-25

**Authors:** Eduard H. J. Voormolen, Peter A. Woerdeman, Marijn van Stralen, Herke Jan Noordmans, Max A. Viergever, Luca Regli, Jan Willem Berkelbach van der Sprenkel

**Affiliations:** 1 Department of Neurosurgery, University Medical Center Utrecht, Utrecht, The Netherlands; 2 Image Sciences Institute, University Medical Center Utrecht, Utrecht, The Netherlands; 3 Department of Medical Technology and Clinical Physics, University Medical Center Utrecht, Utrecht, The Netherlands; Harvard Medical School, United States of America

## Abstract

**Background:**

A neuronavigation interface with extended function as compared with current systems was developed to aid during temporal bone surgery. The interface, named EVADE, updates the prior anatomical image and visualizes the bone drilling process virtually in real-time without need for intra-operative imaging. Furthermore, EVADE continuously calculates the distance from the drill tip to segmented temporal bone critical structures (e.g. the sigmoid sinus and facial nerve) and produces audiovisual warnings if the surgeon drills in too close vicinity. The aim of this study was to evaluate the accuracy and surgical utility of EVADE in physical phantoms.

**Methodology/Principal Findings:**

We performed 228 measurements assessing the position accuracy of tracking a navigated drill in the operating theatre. A mean target registration error of 1.33±0.61 mm with a maximum error of 3.04 mm was found. Five neurosurgeons each drilled two temporal bone phantoms, once using EVADE, and once using a standard neuronavigation interface. While using standard neuronavigation the surgeons damaged three modeled temporal bone critical structures. No structure was hit by surgeons utilizing EVADE. Surgeons felt better orientated and thought they had improved tumor exposure with EVADE. Furthermore, we compared the distances between surface meshes of the virtual drill cavities created by EVADE to actual drill cavities: average maximum errors of 2.54±0.49 mm and −2.70±0.48 mm were found.

**Conclusions/Significance:**

These results demonstrate that EVADE gives accurate feedback which reduces risks of harming modeled critical structures compared to a standard neuronavigation interface during temporal bone phantom drilling.

## Introduction

Surgical approaches through the temporal bone require some form of temporal bone drilling to create an adequate access towards the surgical target. Skull base surgeons need to be thoroughly oriented during temporal bone drilling to optimize access creation while minimizing bone removal and evading critical structures, such as the facial nerve and sigmoid sinus. Anatomical landmarks are the traditional means of orientation during temporal bone drilling; however, these are subject to high inter-individual variability [1] and can be eroded by tumor, inflammation or previous surgery. Neuronavigation (i.e. frameless image guidance) techniques offer surgeons alternative modern means of intra-operative orientation during temporal bone surgery [2], [3], [4], [5], [6], [7], [8].

Neuronavigation systems display the location of the tip of a tracked drill on a navigation map of the patient's anatomy imaged pre-operatively. Contemporary neuronavigation systems offer ‘point in space’ feedback, which has limitations: The navigation scan is not updated while the patient's anatomy is altered by drilling, so the surgeon remains visually uninformed in regards to the relationship of the size of the surgical approach as compared to the size underlying tumor. Furthermore, standard neuronavigation does not adequately notify the surgeon about where he/she is drilling in relation to surrounding temporal bone critical structures.

**Figure 1:**
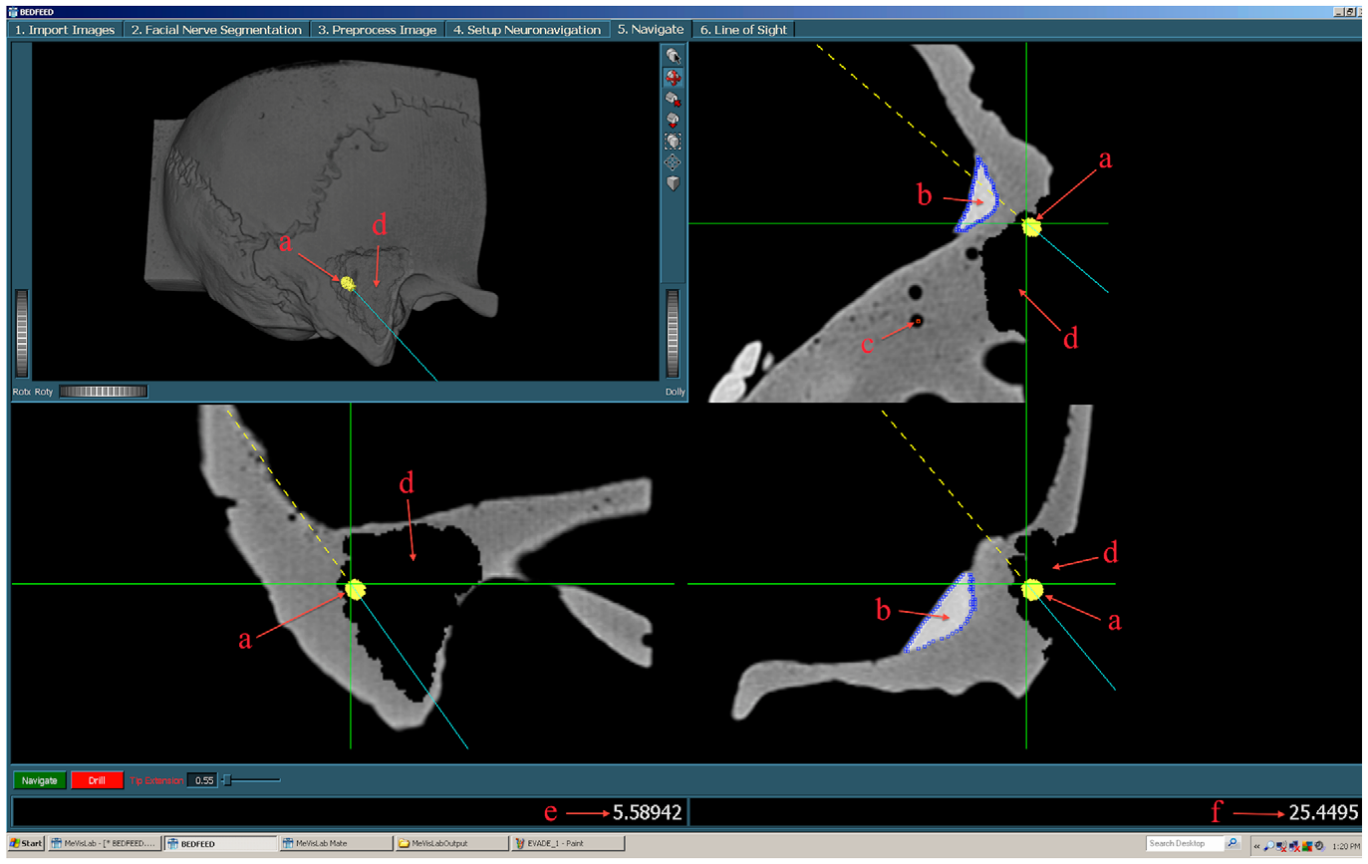
The EVADE Interface. The EVADE interface is shown. Figure annotations (a–f) are displayed in red. In the upper left corner a 3D rendering of the anatomy (in this case a temporal bone phantom) is shown and around it are three orthogonal sections. The green cross designates the current position of the drill tip. The yellow shape represents the drill bit being used (a). The modeled sigmoid sinus (b) and facial nerve (c) are outlined in blue and orange respectively. The virtually drilled cavity is displayed both in 3D as in 2D (d). The two numbers on the bottom give the current distance to the sinus (e) and facial nerve (f).

In order to improve these aspects, we designed and implemented a novel neuronavigation interface that augments the information relay to the surgeon (Figure 1). Our interface has two special characteristics: First, it shows the bone drilling process virtually in real time, providing feedback on the entire progress of bone drilling. So, the surgeon can see the extent of his drill cavity at all times. Second, it allows (semi-automatic) segmentation of temporal bone critical structures (such as the facial nerve [9], [10]) and continuously updates the distance of the tracked drill to these structures and emits audiovisual warnings [11] when the drill tip comes in (too) close proximity. Our interface is referred to as EVADE: ‘Exposure Visualization and Audible Distance Emission’ (Video S1).

Here we evaluate the accuracy and surgical utility of EVADE in phantom models. The aim of this article is threefold. First, we assess whether it is possible to track a drill tip with sufficient accuracy. Second, it is investigated whether EVADE is able to show virtual bone excavation truthfully. Third, we conduct a trial to test EVADE's added surgical value by comparison with a standard neuronavigation system.

## Results

The accuracy of tracking a drill tip while navigating a cylinder and ball phantom was assessed by measuring the target registration error (TRE). The mean TRE was 1.33±0.61 mm (Table 1). The maximum TRE measured was 3.04 mm, which was obtained with a 5 mm drill bit.

**Table 1:** Drill Tracking Accuracy Results.

Experiment	Pointer	SD	3 mm	SD	4 mm	SD	5 mm	SD
1	0.85	0.48	1.02	0.51	1.13	0.65	1.21	0.59
2	1.13	0.33	1.59	0.33	1.47	0.67	1.26	0.56
3	1.25	0.46	1.75	0.51	1.32	0.52	1.92	0.59
4	-	-	1.19	0.53	0.85	0.40	1.64	0.53
Average (mm)	1.05	0.45	1.36	0.57	1.17	0.59	1.47	0.63
Maximum (mm)	2.34		2.83		3.01		3.04	

This table displays results for four separate tracking accuracy experiments on the cylinder and ball phantom in the operating room. Average target registration errors are given in millimeters for the “Pointer” and a drill with “3 mm”, “4 mm” or “5 mm” drill bits attached, for each experiment. Additionally, overall average and maximum target registration errors for each of the instruments are displayed in millimeters in the row “Average” and “Maximum” respectively. “SD” means standard deviation.

**Figure 2:**
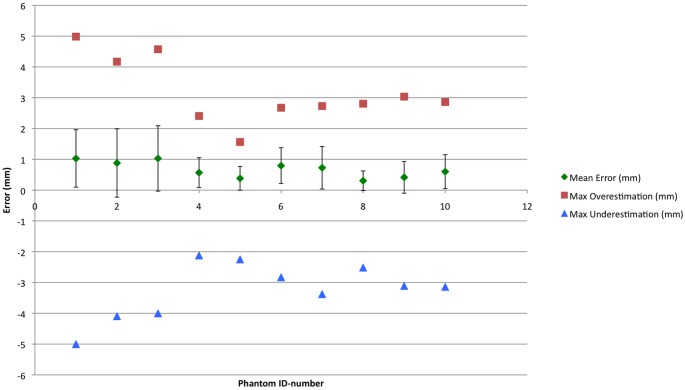
Exposure Visualization Accuracy Results. The mean and maximum over- and underestimation errors in virtually representing the drill cavity (i.e. the surgical exposure) are presented in millimeters (on the y-axis) for each temporal bone phantom (whose index is displayed on the x-axis). Note the differences in errors between the first three models and the last seven models in which higher resolution CT scans were used.

**Figure 3:**
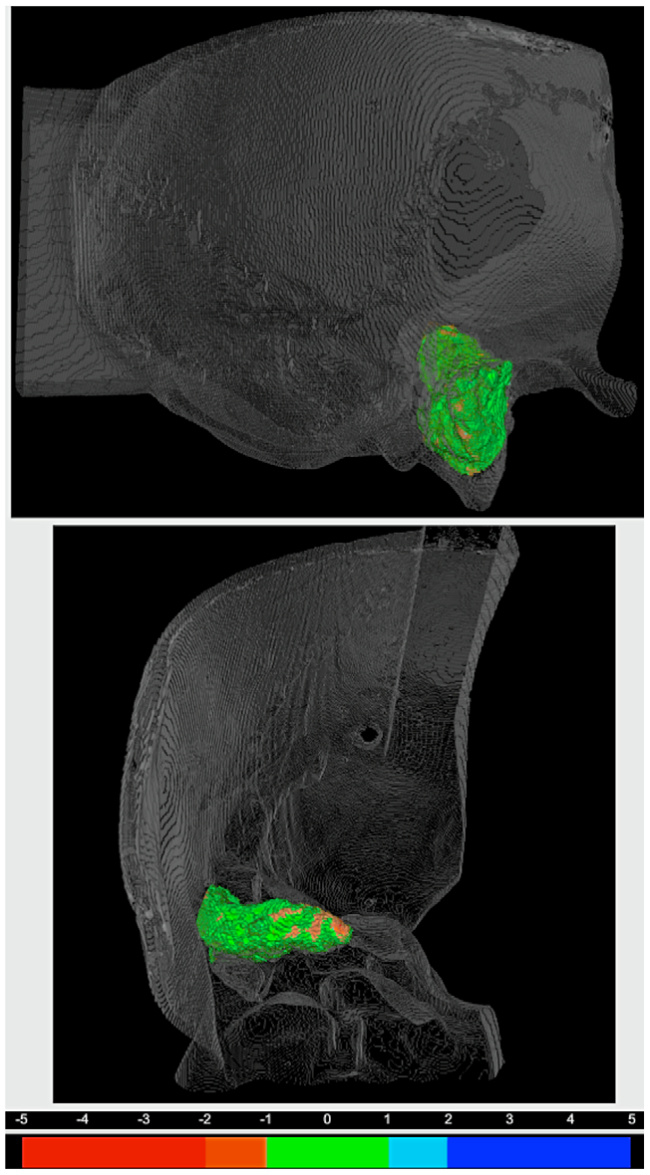
Illustration of Drill Cavity Overlap Error. The error-to-color coded surface to surface distance map of drill cavity index number 6 is displayed in a 3D rendering of the corresponding temporal bone. The top view is from lateral and the bottom view shows the cavity from anterior. The legend for the error-to-color representation is provided under the 3D renderings. Note the green areas within the distance map denote errors of under 1 mm, and the orange areas represent virtual underestimation errors of between 1 and 2 mm.

Next, it was investigated how accurate the EVADE interface could virtually depict the drilling process (Figure 2). All five neurosurgeons had the qualitative impression during surgery that the displayed virtual drill cavity was correct. The average real-to-virtual drill cavity overlap, i.e. surface-to-surface distance (mean surface-to-surface distance averaged over ten temporal bone models), measured 0.67±0.66 mm. The average virtual maximum overestimation and underestimation was 3.25±0.91 mm and 3.18±1.06 mm respectively. An error-to-color coded map of a drill cavity is provided in Figure 3. Subgroup analysis showed that for the first three models (indices 1–3) the average mean surface-to-surface distance was 0.98±0.09 mm with maximum over- and underestimations of 4.58±0.41 mm and 4.37±0.55 mm. The last seven models (indices 4–10), which were imaged at higher resolution, showed an average mean surface-to-surface distance of 0.53±0.18 mm with 2.54±0.49 mm and 2.70±0.48 mm over- and underestimation respectively.

Furthermore, a trial was performed that compared the clinical efficacy of a standard navigation interface vs. the EVADE interface during temporal bone phantom drilling. While using the standard navigation interface the average fiducial registration error (displayed on the navigation machine) was 0.70±0.14 mm. The surgeons in this group required an average of 33±11 minutes to perform the surgery. The modeled facial nerve was hit on two occasions. Once by an experienced staff surgeon and once by a resident. The modeled sigmoid sinus was damaged once by a staff neurosurgeon. The average qualitative scores for surgeon satisfaction with intraoperative orientation and the resulting exposure were 3.6/5.0 and 2.8/5.0 respectively. During usage of EVADE navigation the fiducial error registration error was 0.82±0.18 mm. The average time required for exposure was 31±7 minutes. The modeled facial nerve and the sigmoid sinus were not hit by any surgeon. The scores for satisfaction with intra-operative orientation and resulting exposure with EVADE were 5.0/5.0 and 4.4/5.0 respectively. Results of statistical comparisons between trial groups were deemed unreliable because of small sample size and therefore were not included.

## Discussion

The purpose of EVADE is to augment standard neuronavigation adding audiovisual feedback to further aid the surgeon in performing trans-temporal surgery. The absence of soft-tissue shift in the temporal bone makes it possible to maintain high spatial tracking accuracy of a tracked drill throughout the whole approach.[12], [13] To the best of our knowledge we are the first to confirm that a commercial neuronavigation system can indeed track a drill with attached tracking frame at high accuracy on a high resolution CT image, with corresponding mean TRE of 1.3 mm and maximum TRE of 3 mm. We believe this accuracy to be sufficient for temporal bone neuronavigation since the error is not larger than the average distance between temporal bone critical structures (i.e. the area that needs to navigated).

**Figure 4:**
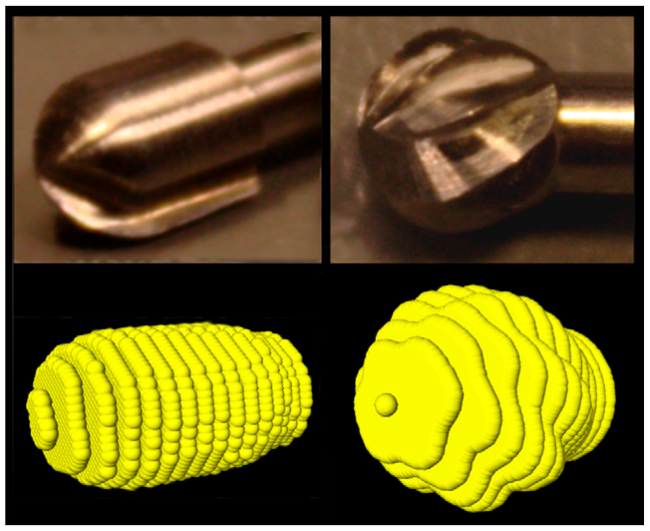
Virtual Drill Bits. Drill bits were scanned with high resolution CT and represented as 3D point clouds. On the left is displayed a 3 mm match-head drill bit and on the right a 4 mm drill bit can be seen.

EVADE harnesses drill tracking information to give online intra-operative image updates of the drill cavity without the need for intra-operative imaging and associated radiation. Previous work concerning the use of navigation interfaces with such ‘exposure visualization’ features has been done by Wurm et al. (2008) [14] and at our institute by Woerdeman et al. (2009) [15]. Like EVADE, these neuronavigation feedback modes adjust voxel intensities around a tracked instrument tip. The difference is that EVADE uses geometric models of drill bits to erase voxels to simulate drilling, while the earlier encompassed simple spheres. The major problem of simulating drilling with spheres is its inherent inaccuracy should the surgeon use non-spherical drill bits (such as the 3 mm match head drill bit in Figure 4). In such case, we postulate that the drill cavity will not be represented truthfully. Instead, when using geometric model (in the exact shape of the non-spherical drill bit) for voxel erasing the accuracy of depicting the drill cavity is improved.

Here, we demonstrate that the EVADE interface truthfully represents surgeon-made drill cavities, with maximum errors of approximately 3 mm.

In general, neuronavigation tracking errors can be caused by inaccuracy in:

1. designating fiducial points in the image
2. designating fiducial points on the patient (or phantom)
3. patient-to-image fiducial point calculations
4. measuring the tracking frame position in space
5. drill tip-tracking frame calibration errors

The above sources of inaccuracy cause the errors observed in the cylinder and ball phantom experiments.

Furthermore, the implementation of EVADE's virtual drilling adds more sources of imprecision due to:

1. drill cavity sampling due to (low) image resolution
2. modeling of the drill bit geometry

We investigated whether these additional sources of inaccuracy contribute to the total neuronavigation error in the temporal bone phantom experiments. The location of neuronavigation errors were visualized on 3D error-to-color coded surface maps. In the first three phantoms, we observed that errors were systematically largest along the axis of lowest image resolution (the z-direction). These errors were caused by drill cavity sampling inaccuracy. Therefore, we changed the imaging protocol to isotropic scans with a higher z-resolution. Consequently, in the last seven models such a distinct error pattern was not identified and consistently lower error values were observed. These error values corresponded to the errors we found during the cylinder and ball phantom experiments. So, we can conclude that modeling of the drill bit geometry does not contribute to the neuronavigation error. Moreover, we stress the importance of using EVADE with an isotropic high resolution image to improve designation of fiducials in the image and drill cavity sampling.

The error color-coded maps show the location of signed errors (Figure 3). Positive errors represent areas where the EVADE interface overestimates the size of the drill cavity. Overestimation errors cause the system's monitor to display particular anatomy as absent, while it is still present within the operating field. This may lead the surgeon to mistrust and eventually discard the system.

Conversely, negative errors represent ‘under-estimation’ errors of the drill cavity. Underestimation errors could potentially be dangerous since the surgeon gets the impression from the system's monitor that he/she might drill further to arrive at a particular target while in fact it has already been reached. In a worst-case scenario, erroneous drill cavity underestimation might contribute to iatrogenic injury of temporal bone critical structures.

**Figure 5:**
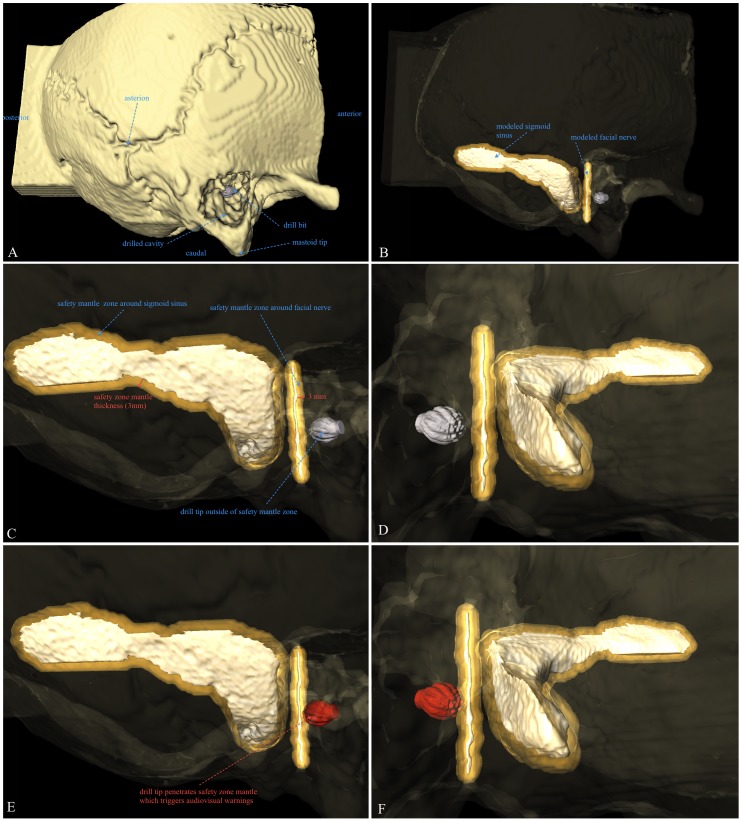
Illustration of Critical Structure Safety Mantle Implementation. This figure illustrates the principle of the critical structure safety mantle implementation which EVADE uses to generate timely audiovisual warnings in spite of drill tracking inaccuracies of the navigation machine. Figure 5A shows the temporal bone phantom for purposes of anatomical orientation. In figure 5B the bone phantom has been rendered translucent to show the drill bit (in grey) and modeled critical structures; the sigmoid sinus and the facial nerve. Figure 5C is a zoomed in view on the critical structures (in white) in which the safety mantle (the orange-golden translucent area) is visible around the critical structures. Note that the safety mantle thickness measured from the surface of the structures is 3 millimeters. Figure 5D shows the same situation from a different angle. The drill bit is still outside of the safety mantle. In Figure 5E the surgeon has continued drilling and the drill bit tip (now in red) has entered the safety mantle around the facial nerve. EVADE is triggered to provide audiovisual warnings. Figure 5F shows the situation as in 5E from a different angle.

EVADE was primarily designed to prevent such adverse events by its audible distance warning mechanism (see Methods section; Audible Distance Emission Implementation). The special attribute of this warning mechanism is the safety mantle imposed around critical structures (Figure 5). The thickness of the safety mantle can be adjusted to compensate for drill tracking errors. [9]. Therefore, this safety mantle implementation uncouples the magnitude of the position tracking error from the size of segmented critical structures. Even if the tracking error is larger than the critical structure size, EVADE still gives timely audiovisual warnings. Our phantom results indicate that for this to work properly the safety mantle thickness should be 3 mm when using optical tracking, high resolution isotropic CT images and a drill with attachable tracking frame. We wish to validate this safety mantle thickness value further in the context of real human temporal bone anatomy with realistic critical structures. Therefore, cadaver head experiments are currently being performed. These experiments are part of the second and final pre-clinical phase, after which EVADE will be ready for testing in patients.

It is important to emphasize that audible distance emission will work only if temporal bone critical structures have been delineated accurately in individualized pre-operative images. EVADE incorporates a semi-automated method to segment the facial nerve in CT scans of patients (NerveClick) [9], [10]. We are currently developing algorithms for automated segmentation of other temporal bone structures. However, these algorithms are tuned to find structures within images of patients. Since the phantoms had very different image characteristics compared to patients, we could not use these segmentation algorithms for this study. Instead, we used manual segmentation to designate the positions of modeled critical structures within each individual phantom's image (see Methods section; Critical Structure Segmentation).

The results of the interface trial indicate that EVADE reduces the risks of iatrogenic injury to critical structures and improves the intra-operative surgical orientation and exposure of the tumor in comparison to a standard neuronavigation interface.

Note that temporal bone drilling was conducted on phantoms which had far less bony landmarks for surgical orientation than a real temporal bone. Moreover, the modeled critical structures just approximated the shapes of actual temporal bone structures. So, instead of relying on anatomical knowledge the surgeons had to depend heavily on the feedback received from the neuronavigation interface to find a safe approach to the tumor.

The disadvantage of the phantom design is that it hampers surgical realism. Therefore, the trial results do not necessarily forebode that EVADE will improve surgery on actual patients. On the other hand, the phantom design does allow for testing the surgical usefulness of the navigation information (i.e. the amount of anatomical insight) provided during surgery. Therefore, the trial results demonstrate that EVADE is a superior surgical navigation interface as compared to the current standard interface. We anticipate that EVADE will aid the surgeon in difficult clinical cases with aberrant temporal bone anatomy due to extensive pathology or prior surgery. In such cases, it is our experience that neither bony landmarks nor conventional neuronavigation provide enough information for accurate surgical orientation.

Besides the phantom design, this study has several other limitations. The sample size for the interface trial was small and rendered statistical analyses unreliable. Therefore, we did not include statistical test results. Another disadvantage that impedes extrapolating the trial results to the actual clinical situation was that not all trial surgeons were experienced skull base surgeons. Interestingly, two of three critical structures were hit by an experienced skull base surgeon (using a standard neuronavigation setup).

In conclusion, our results demonstrate that the EVADE neuronavigation interface is accurate. Furthermore, we show that EVADE's intra-operative feedback reduces risks of harming modeled critical structures compared with using a standard neuronavigation interface during trans-labyrinthine surgery of temporal bone phantoms. Further pre-clinical validation of EVADE in cadaver heads is necessary to confirm that the technical benefits observed in the present phantom study can be extended to patients receiving temporal bone surgery.

## Materials and Methods

### Hardware

The EVADE system's hardware consists of a Stealth Treon navigation machine (Medtronic Inc. Boulder CO, USA) used for its optical tracking capabilities and patient-to-image registration algorithm, and a separate laptop computer (Apple Inc. Cupertino CA, USA) running Windows XP (Microsoft Corp. Redmond WA, USA) connected via a network cable. The laptop outputs its display to a 21.3″ sized display monitor. A SureTrak™ frame (Medtronic Inc. Boulder CO, USA)) was attached to the drill allowing the navigation machine to track it. The phantoms were fixed to the operating table with a Mayfield head clamp (Integra LifeSciences Corp. Cincinnati OH, USA). A reference frame (Medtronic Inc. Boulder CO, USA) was attached to the Mayfield clamp to translate drill coordinates recorded in camera space to coordinates in image space.

### Software

The commercial software StealthLink (Version 1.0, Medtronic Inc. Boulder CO, USA) was used to interface between the navigation machine and our custom made software (build with MeVisLab Programming Environment 2.0, MeVis Research, Bremen, Germany; www.mevislab.de. The necessary custom made software modules are available at request from the principle author) running on the laptop computer. Drill tip and hind positions and resulting drill shaft orientations were calculated (in image space) on the laptop computer from information provided via StealthLink.

### Drill Calibration

The system needs to know the relation between the tracking frame and the tip and hind of the drill to calculate the image space positions. Therefore, it needs to be calibrated before surgery. The calibration procedure involves three steps: First, the pointer is placed into a divot within the reference frame with its shaft parallel to the long axis of the divot. Second, the drill is placed within the same divot with its shaft positioned analogously to the pointer in the previous step. Third, the drill is placed next to the divot directly on the reference frame while keeping its shaft in the same orientation as during the previous step. This is to compensate for the fact that some drill bits are large and cannot reach the bottom of the divot. Effectively, their tip does not reach the exact location where the pointer tip was located during the first calibration step, which leads to inaccuracies. To adjust for this, the difference in drill tip distance along the drill's shaft between being in the divot and just next to the divot is calculated. Subsequently, the difference between the drill tip distance and the divot depth is added to the tip of the drill. The drill hind is calculated to be at a fixed point 10 cm above the drill tip along the drill shaft.

### Phantoms

**Figure 6:**
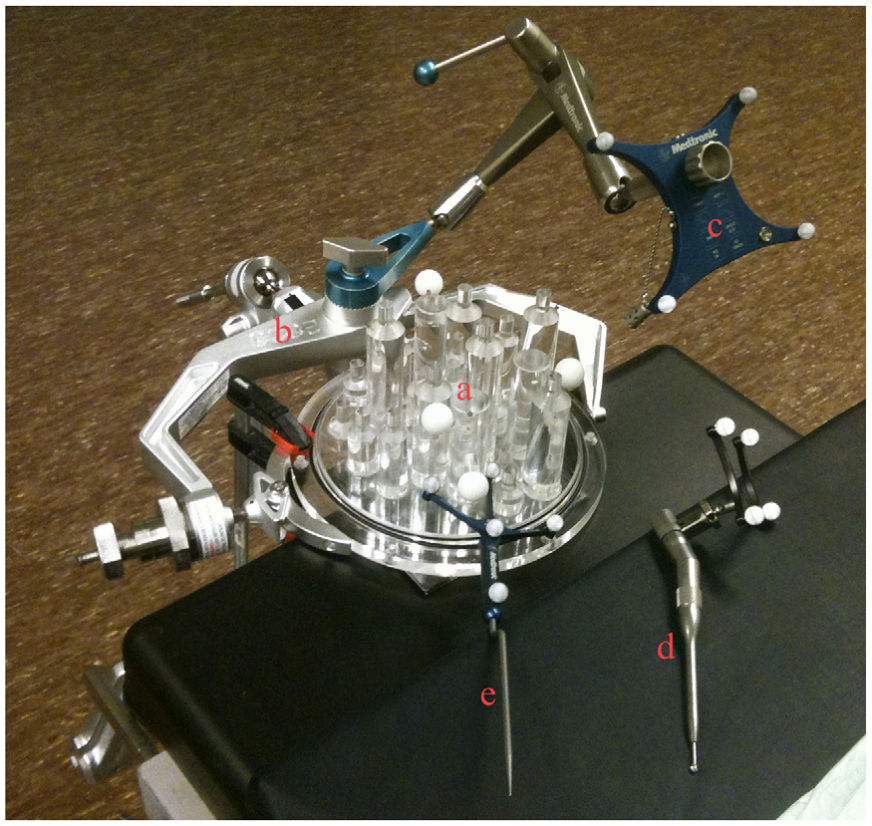
Drill Tracking Accuracy Experimental Setup. The setup in the operating room during drill tracking accuracy experiments on the cylinder and ball phantom (a) is shown. Note the head clamp (b) and reference frame (c). Registration of the phantom was performed via four rigidly attached screws that served as fiducial markers. The top of the cylinders were touched with the drill (d) with attached tracking frame and pointer (e) and the image coordinates were recorded and compared with the actual positions to yield target registration errors.

Two different phantoms were used for our experiments. A cylinder and ball phantom (Figure 6) was used to assess the accuracy of tracking a drill. The phantom consisted of 19 cylinders of different lengths spread across its base, on top of which hollow balls could be placed. The centers of these balls correspond to the top center points of the cylinders, which locations are designated with a small divot. The phantom was fitted with four metal screws to serve as fiducial markers.

**Figure 7:**
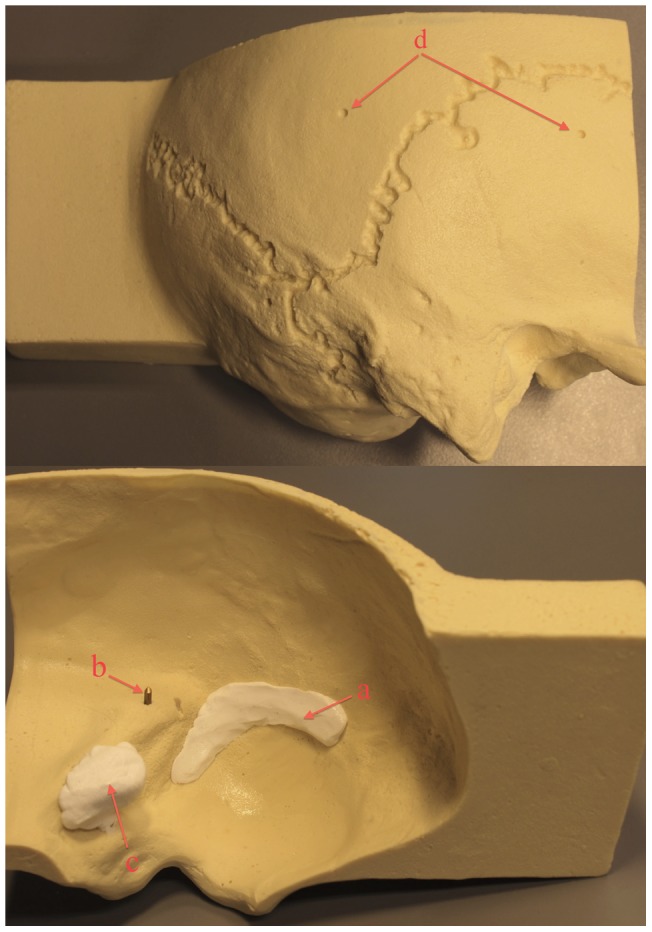
Temporal Bone Phantoms. This figure shows an example of a plastic temporal bone phantom. On the outside divots (d) were drilled to be used as fiducial markers for registration. On the inside a modeled silicon sigmoid sinus (a) and tumor resembling a vestibular schwannoma (c) were placed. Also, a straight canal was drilled in which a metal rod was placed serving as a modeled facial nerve (b).

Furthermore, we used temporal bone phantoms constructed from drillable plastic (Sawbones Europe AB, Malmö, Sweden). In each model a straight canal was drilled by hand to resemble the mastoid section of the facial nerve canal and silicon gel was applied on the intra-cranial side to model the sigmoid sinus and a vestibular schwannoma tumor (Figure 7). This ensured that each model was slightly different from the next. Six divots were drilled into each of these models to serve as fiducial markers.

### Scan Parameters

The cylinder and ball phantom was scanned on a 64-slice Philips CT scanner. Scan parameters were set to 120 kVp and 200 mAs, which yielded images with a matrix size of 512×512×207 with voxels of 0.48×0.48×1.0 mm3. The temporal bone phantoms were scanned on either a 64- or 256-slice Philips CT scanner. Two different protocols were used. For models 1–3 we scanned with 120 kVp, 300 mAs acquiring images with a matrix size of 712×712×168 and anisotropic voxel sizes of 0.18×0.18×1.0 mm3. Models 4–10 were scanned with the following parameters: 120 kVp, 400 mAs, matrix size of 512×512×281 with voxel sizes of 0.34×0.34×0.4 mm3. All models were re-scanned post-operatively using the same scan protocol as pre-operatively.

### Exposure Visualization Implementation

EVADE's virtual drilling relies on knowledge of the drill tip location and the orientation of its shaft which information is acquired approximately every 0.16 seconds through StealthLink. The drill bit is represented as a collection of points (3D point cloud) sampled from a prior ultra-high resolution CT image of the drill bit with matrix sizes of 768×768×45 with voxel sizes of 0.096×0.096×0.35 mm3. We constructed point clouds of 3, 4 and 5 mm drill bits (Figure 4). Within these point clouds the tip point and hind point were designated to be aligned with the drill's axis shaft. The point clouds were of higher resolution than the phantom's CT images. Every point is interpolated to the closest voxel through nearest-neighbor interpolation. Subsequently, these voxels are accessed and their voxel value is set to match the background (air) intensity. A drill bit shape is effectively ‘removed’ from the model's image. During surgery, many consecutive drill tip position updates create a virtual drill cavity within the model's image.

### Critical Structure Segmentation

To make audible distance emission work the system needs to learn the image positions of critical structures. Therefore, these structures were designated on individual images of the phantoms acquired pre-operatively via manual segmentation: it required the surgeon to draw contours around the structures slice-by-slice. Subsequently, 3D volumetric images of the structures were generated by adding all contours. The 3D volumes were transformed into point clouds by sampling the surfaces at a resolution of 0.1 mm. In this way, EVADE learned the position of the facial nerve and sigmoid sinus for each phantom.

### Audible Distance Emission Implementation

EVADE's audible distance emission feature works as follows: the system calculates the Euclidian distance from the drill bit tip coordinate to the closest points on the critical structure point clouds continuously. If this distance becomes less than a particular predefined distance, known as the safety mantle thickness, it gives off a distinct audiovisual warning notifying the surgeon that he/she is drilling in (too) close proximity of the critical structure. So effectively, a safety mantle that follows the contours of the segmented critical structures is imposed, and EVADE tracks the drill tip continuously during drilling to warn when the drill tip penetrates this safety mantle (Figure 5) [9]. The surgeon hearss the warning without having to discontinue drilling to look at the monitor, and can take appropriate actions (e.g. release pressure on the drill, drill in a different direction, change the drill bit, etc.). The thickness of the safety mantle determines how ‘early’ EVADE produces warnings. All surgeons used the same safety mantle thickness of 3 mm.

### Experiment Protocols

Two different experiments were performed. First, we assessed how accurate the EVADE system could track the drill tip on the cylinder and ball phantom. A high resolution CT image of the phantom was acquired, after which the phantom was taken to the operating room, placed in a Mayfield head clamp and registered. Subsequently, the drill tip was positioned at the small divots at the top center points of the 19 cylinders, and the 19 image positions were saved. This experiment was conducted four times (each instance requiring a new setup and registration) using 3, 4, and 5 mm cutting drill tips, amounting to a total of 228 measurements. We also acquired image positions for the standard navigation pointer to obtain a reference accuracy measure.

**Figure 8:**
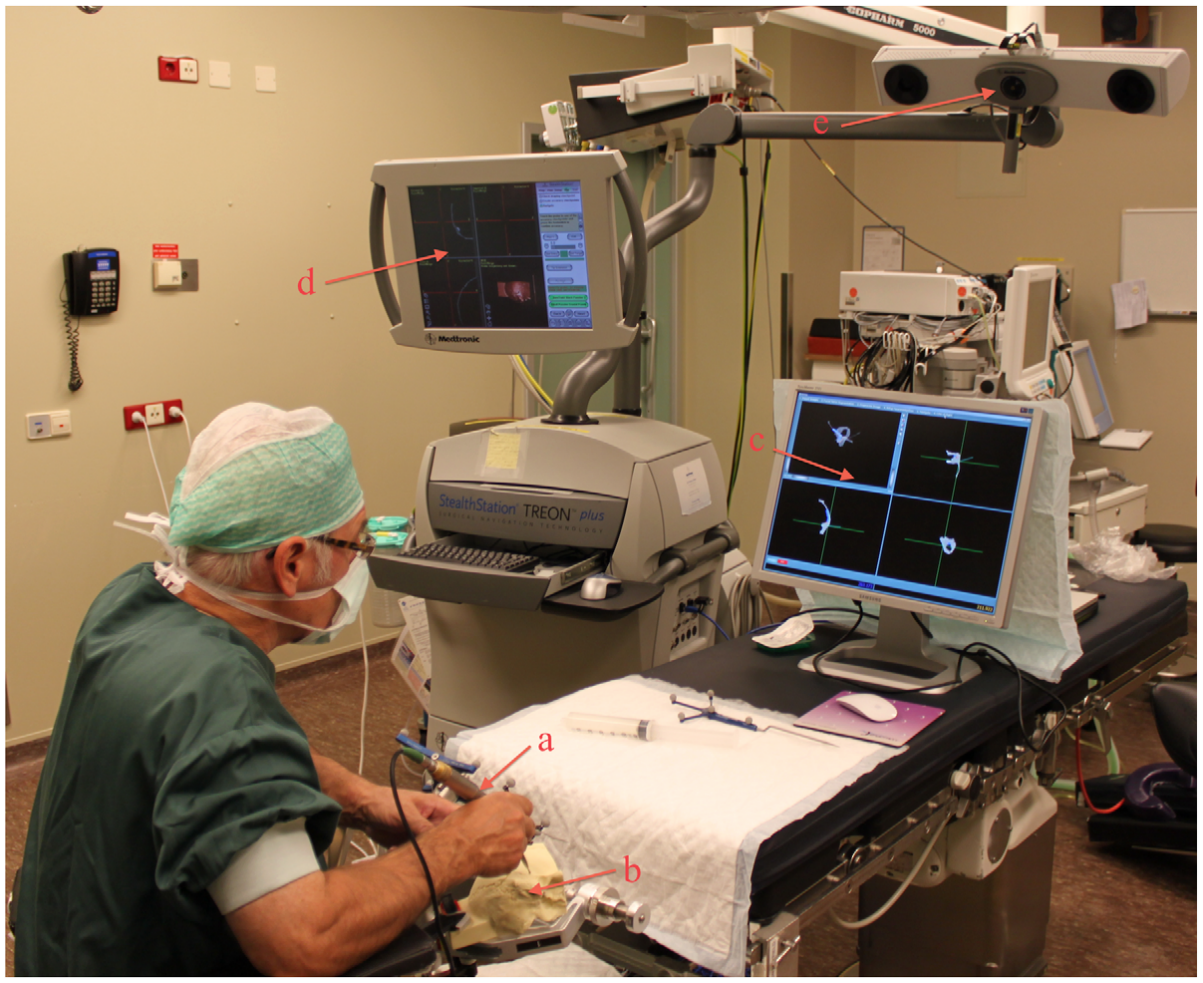
Intra-Operative Setup during Temporal Bone Surgery. This figure shows the typical situation during a trans-labyrinthine approach with a navigated drill (a) on the temporal bone phantoms (b) in the operating room. The surgeon used either the EVADE interface (c) or the standard navigation interface (d). Note the infra-red camera (e) used for tracking.

Second, ten temporal bone phantoms were scanned with high-resolution CT. The modeled facial nerve canal and sigmoid sinus were segmented. Subsequently, the phantoms were taken to the operating room, placed in a Mayfield headclamp and registered (Figure 8). The fiducial registration error calculated by the neuronavigation system was stored. Five different neurosurgeons were asked to each perform a trans-labyrinthine approach to the modeled vestibular schwannoma on two phantoms, for the interface trial comparing EVADE to a standard navigation interface. In half of the cases the surgeons were exposed to the augmented feedback EVADE offers (i.e. real time drill cavity updates and distance feedback with audible warnings of the modeled facial nerve and sigmoid sinus) and in the other half they used standard navigation while EVADE was running silently in the background (calculating a virtual drill cavity). The order of whether or not EVADE was used, was decided randomly. Time between the first and second surgery was on average 260±177 days. The surgeons used one drill bit per surgery. The virtually drilled image of the temporal bone phantom created by EVADE was saved after the surgeons stopped drilling. The decision to stop surgery was made by the surgeons. They were instructed to stop once they thought they had achieved their best exposure of the modeled tumor. The drilled phantoms were re-scanned post-operatively with high resolution CT.

### Data Analysis

Target registration errors (TRE) of tracking a drill with attached SureTrak™ on the cylinder and ball phantom were calculated in the follow way. We obtained the true image position of the top center of the cylinders via image analysis on the model's CT image: each ball, positioned on top of one of the nineteen cylinders, was segmented (using a 3D region growing algorithm) and its center of mass was calculated which corresponded to the true image position of the cylinder top center. The TRE was calculated as being the Euclidian point-to-point distance between the true image positions of the cylinder top center and the measured image position while the drill was touching that cylinder's top center divot. Obtained TREs were averaged to yield the main outcome measure for this experiment: mean TRE.

Furthermore, we performed image processing to compare images of the temporal bone model drill cavities virtually ‘erased’ by EVADE to images of the corresponding real drill cavities. For each temporal bone model the post-operative CT image of the drilled model was registered globally with a fully automated mutual-information based affine registration algorithm to its original CT image [16]. The virtually drilled model was not registered because its world matrix (i.e. its scaling, position, and orientation) was identical to the original model image. Both virtual and real drilled model images were subtracted from the original model image. Drill cavities were segmented in the subtraction images using a 3D region growing algorithm to obtain images of the virtual and real drill cavity. The virtual and real cavity images were overlaid and converted to 3D surface meshes without loss of resolution (i.e. with nodes at every voxel). The region of the cavity surfaces corresponding to the area where the surgeon started drilling on the outer surface of the temporal bone phantom was excluded from analysis. Inclusion would bias results because here correspondence between cavities was optimal. The mean and signed maximum Euclidian surface-to-surface distances between the real and virtual drill surface were calculated.

The resulting surface-to-surface distances were a measure of the virtual drilling error: if the distance is zero, there is perfect overlap and the virtual drilling corresponds exactly to the real drilling. If the distance is non-zero, EVADE either overestimated or underestimated the cavity compared to reality. To visualize the location and magnitude of the virtual drilling errors, and to depict areas of over- and underestimation, 3D error-to-color coded surface maps were generated (Figure 3).

### Trial Neurosurgeons

Five different trial surgeons participated in the interface trial. Three surgeons were neurosurgical staff members with extensive experience in skull base surgery (over fifty approaches) and two were neurosurgical residents who had participated in five or less skull base approaches.

### Trial Outcome Measures

Four outcome measures were acquired for the interface trial. The surgeons impressions of the navigation system were noted via a standardized questionnaire. Two questions were asked: 1) How satisfied are you with the exposure of the tumor? 2) How well do you think your surgical orientation was during surgery? The questionnaire allowed answers to be given on a five point scale with 1 reflecting a very poor verdict and 5 an outstanding verdict. The surgeons used common sense, their clinical training and experience to form an opinion of the surgical exposure of of the modeled tumor.

Furthermore, the phantoms were assessed visually post-operatively for damage to the modeled facial nerve and sigmoid sinus. We also measured the time required by the surgeon to perform a satisfactory exposure.

## Supporting Information

Video S1 **Demonstration of EVADE**'**s novel information feedback characteristics.** This video shows surgeons performing a trans-labyrinthine craniotomy on a temporal bone phantom while using EVADE neuronavigation. It provides an illustration of how the ‘exposure visualization’ and ‘audible distance emission’ features of the interface can be used in the operating theatre.
(MOV)

## References

[1] Gharabaghi A, Rosahl SK, Feigl GC, Liebig T, Mirzayan JM (2008). Image-guided lateral suboccipital approach: part 1-individualized landmarks for surgical planning.. Neurosurgery.

[2] Gharabaghi A, Rosahl SK, Feigl GC, Safavi-Abbasi S, Mirzayan JM (2008). Image-guided lateral suboccipital approach: part 2-impact on complication rates and operation times.. Neurosurgery.

[3] Pillai P, Sammet S, Ammirati M (2009). Image-guided, endoscopic-assisted drilling and exposure of the whole length of the internal auditory canal and its fundus with preservation of the integrity of the labyrinth using a retrosigmoid approach: a laboratory investigation.. Neurosurgery 65: 53–59; discussion 59.

[4] Staecker H, O'Malley BW, Eisenberg H, Yoder BE (2001). Use of the LandmarX trade mark Surgical Navigation System in Lateral Skull Base and Temporal Bone Surgery.. Skull Base.

[5] van Havenbergh T, Koekelkoren E, de Ridder D, van de Heyning P, Verlooy J (2003). Image guided surgery for petrous apex lesions.. Acta Neurochir(Wien).

[6] Nemec SF, Donat MA, Mehrain S, Friedrich K, Krestan C (2007). CT-MR image data fusion for computer assisted navigated neurosurgery of temporal bone tumors.. Eur J Radiol.

[7] Miller RS, Hashisaki GT, Kesser BW (2006). Image-guided localization of the internal auditory canal via the middle cranial fossa approach.. OtolaryngolHead Neck Surg.

[8] Sure U, Alberti O, Petermeyer M, Becker R, Bertalanffy H (2000). Advanced image-guided skull base surgery.. Surg Neurol.

[9] Voormolen EH, van Stralen M, Woerdeman PA, Pluim JP, Noordmans HJ (2011). Determination of a Facial Nerve Safety Zone for Navigated Temporal Bone Surgery. Neurosurgery.. In Press.

[10] Voormolen EH, Stralen van M, Woerdeman PA, Pluim JPW, Noordmans HJ, Wong KHHI, D (2011). Intra-temporal facial nerve centerline segmentation for navigated temporal bone surgery..

[11] Woerdeman PA, Willems PW, Noordmans HJ, van der Sprenkel JW (2009). Auditory feedback during frameless image-guided surgery in a phantom model and initial clinical experience.. J Neurosurg.

[12] Kral F, Riechelmann H, Freysinger W (2011). Navigated surgery at the lateral skull base and registration and preoperative imagery: experimental results.. Arch Otolaryngol Head Neck Surg.

[13] Pillai P, Sammet S, Ammirati M (2008). Application accuracy of computed tomography-based, image-guided navigation of temporal bone.. Neurosurgery.

[14] Wurm J, Bohr C, Iro H, Bumm K (2008). Intra-operative image update: first experiences with new software in computer-assisted sinus surgery.. IntJMedRobot.

[15] Woerdeman PA, Willems PW, Noordmans HJ, Tulleken CA, van der Sprenkel JW (2009). The impact of workflow and volumetric feedback on frameless image-guided neurosurgery.. Neurosurgery.

[16] Klein S, Staring M, Murphy K, Viergever MA, Pluim JP (2010). Elastix: a toolbox for intensity-based medical image registration.. IEEE Trans Med Imaging.

